# Vision transformers for the prediction of mild cognitive impairment to Alzheimer’s disease progression using mid-sagittal sMRI

**DOI:** 10.3389/fnagi.2023.1102869

**Published:** 2023-04-13

**Authors:** Gia Minh Hoang, Ue-Hwan Kim, Jae Gwan Kim

**Affiliations:** ^1^Department of Biomedical Science and Engineering, Gwangju Institute of Science and Technology, Gwangju, Republic of Korea; ^2^AI Graduate School, Gwangju Institute of Science and Technology, Gwangju, Republic of Korea

**Keywords:** Alzheimer’s disease, MCI conversion prediction, vision transformers, sMRI image analysis, deep learning, Alzheimer’s disease diagnosis

## Abstract

**Background:**

Alzheimer’s disease (AD) is one of the most common causes of neurodegenerative disease affecting over 50 million people worldwide. However, most AD diagnosis occurs in the moderate to late stage, which means that the optimal time for treatment has already passed. Mild cognitive impairment (MCI) is an intermediate state between cognitively normal people and AD patients. Therefore, the accurate prediction in the conversion process of MCI to AD may allow patients to start preventive intervention to slow the progression of the disease. Nowadays, neuroimaging techniques have been developed and are used to determine AD-related structural biomarkers. Deep learning approaches have rapidly become a key methodology applied to these techniques to find biomarkers.

**Methods:**

In this study, we aimed to investigate an MCI-to-AD prediction method using Vision Transformers (ViT) to structural magnetic resonance images (sMRI). The Alzheimer’s Disease Neuroimaging Initiative (ADNI) database containing 598 MCI subjects was used to predict MCI subjects’ progression to AD. There are three main objectives in our study: (i) to propose an MRI-based Vision Transformers approach for MCI to AD progression classification, (ii) to evaluate the performance of different ViT architectures to obtain the most advisable one, and (iii) to visualize the brain region mostly affect the prediction of deep learning approach to MCI progression.

**Results:**

Our method achieved state-of-the-art classification performance in terms of accuracy (83.27%), specificity (85.07%), and sensitivity (81.48%) compared with a set of conventional methods. Next, we visualized the brain regions that mostly contribute to the prediction of MCI progression for interpretability of the proposed model. The discriminative pathological locations include the thalamus, medial frontal, and occipital—corroborating the reliability of our model.

**Conclusion:**

In conclusion, our methods provide an effective and accurate technique for the prediction of MCI conversion to AD. The results obtained in this study outperform previous reports using the ADNI collection, and it suggests that sMRI-based ViT could be efficiently applied with a considerable potential benefit for AD patient management. The brain regions mostly contributing to prediction, in conjunction with the identified anatomical features, will support the building of a robust solution for other neurodegenerative diseases in future.

## 1. Introduction

Alzheimer’s disease (AD) is one of the most common causes of neurodegenerative disease affecting over 50 million people worldwide. The structural changes of the brain can be one of the biomarkers for identifying AD patients from normal elderly subjects (Chiba et al., 2009; Scheltens et al., 2016). Because of the accumulation of Aβ and the deposition of hyper-phosphorylated tau protein, the structure in the brain begins to shrink, called brain atrophy, especially in specific regions such as the frontal, and hippocampus. Progression of atrophy is first manifest in the medial temporal lobe and then closely followed by the hippocampus, amygdala, and para-hippocampus. Some studies have suggested that in AD patients, entorhinal volumes are already reduced by 20–30%, hippocampal volumes by 15–25% and rates of hippocampal atrophy in mild AD are 3–5% per year. This cerebral atrophy can be visualized in life with MRI (best with a T1-weighted scan) (Johnson et al., 2012). However, most AD diagnosis occurs in the moderate to late stage, which means that the optimal time for treatment has already passed. Mild cognitive impairment (MCI) is an intermediate state between cognitively normal people and AD patients. It refers to mild impairment of cognitive and memory functions rather than dementia. People with MCI tend to convert to AD at a significantly higher rate than normal people. Typically, there are two subtypes of MCI: non-convert MCI (MCINC), which will not develop to AD, and converted MCI (MCIC), which will progress to AD. Therefore, the accurate prediction in the conversion process of MCI to AD may allow patients to start preventive intervention to slow or stop the progression of the disease.

**Figure 1:**
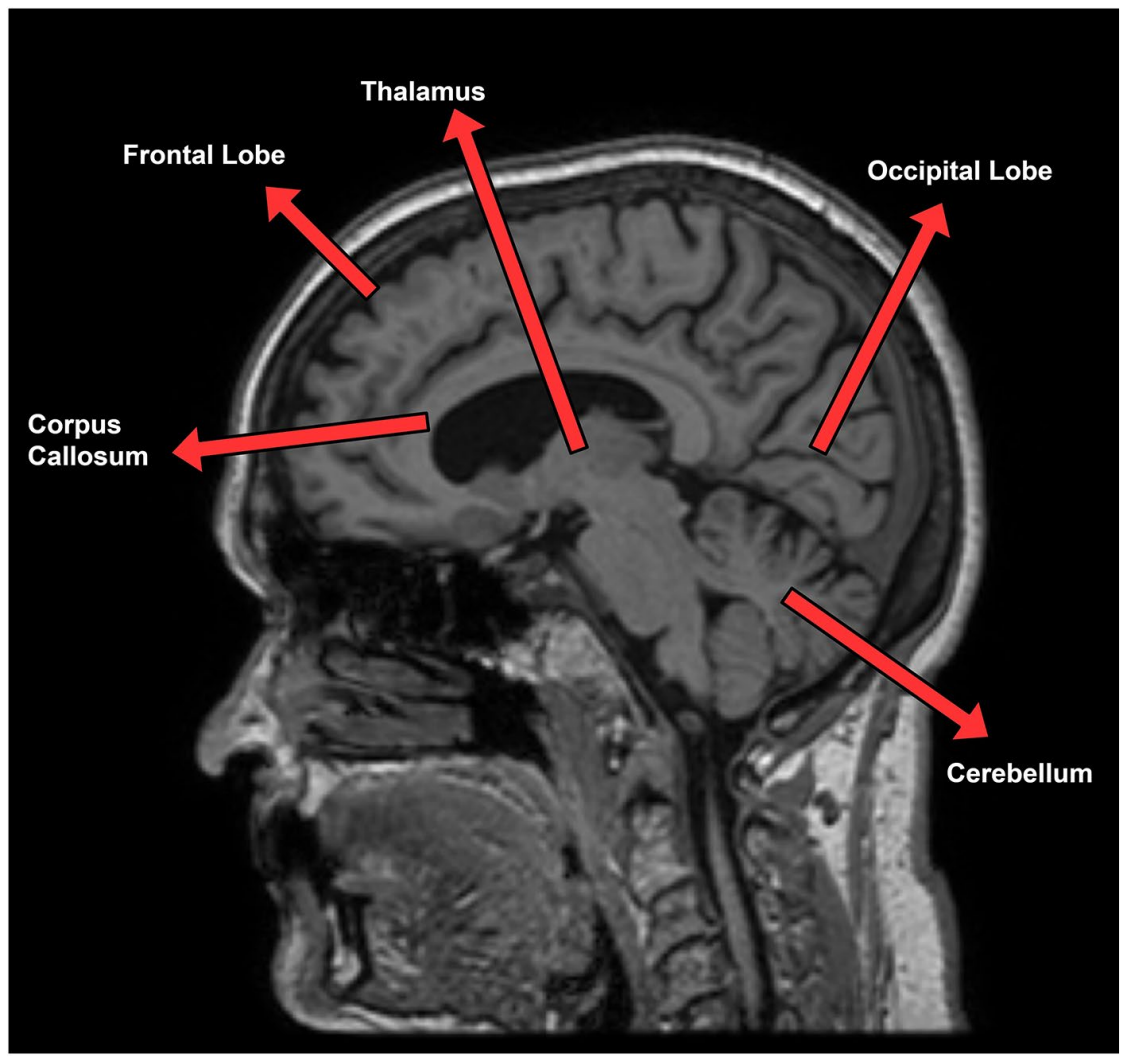
Sagittal plane view of MRI scan taken from ADNI dataset describing AD relevant section of the brain.

As mentioned above, the accumulation of plaque and neurofibrillary tangles make several changes in brain structures. These changes could be used as a biomarker for the classification of MCI progression and are clearly analyzed by structural MRI (sMRI). Three planes of view are there in sMRI known as the axial, sagittal, and coronal planes (Vlaardingerbroek and Boer, 2003). The sagittal plane, especially the mid-sagittal plane provides the most visible information for the diagnosis such as the thalamus, frontal lobe, cerebellum, corpus callosum, which is expected to be the source site for AD tangles and senile plaque. The frontal lobe is in charge of cognitive function in humans and gives an idea about the prognosis of AD (Stuss et al., 1992). Thalamus is also related to episodic memory loss and attention dysfunction in AD (Aggleton et al., 2016). Figure 1 represents a mid-sagittal plane view of an MRI scan taken from the ADNI dataset showing the important section responsible for AD progression. Thus, we adopted the mid-sagittal plane for the assessment of AD for the proposed model.

In recent years, deep learning approaches and their variants have been increasingly applied to AD diagnosis (Islam and Zhang, 2017; Maqsood et al., 2019; Mehmood et al., 2020; Lim et al., 2022). AD classification has been greatly improved by structural MRI-based approaches using the whole brain, image patches, and regions of interest (ROIs). In regional feature-based methods, recent studies mainly relied on prior knowledge to determine ROIs. Using an ROI-based level model, Zhang et al. (2011) achieved an accuracy of 93.2% in AD classification, and 76.4% in MCI classification. Liu et al. (2016) proposed a relationship-induced multi-template learning method for the automatic diagnosis of Alzheimer’s disease based on multiple sets of regional gray matter density features with an accuracy of 93.06% in the AD classification task and 79.25% in the progression MCI task. However, identify and segmenting regions of interest (ROIs) was a time-consuming process that relied on the expertise of specialists, and the features extracted from these regions might not capture the intricate alterations that occur in the brain.

To overcome this limitation, image patch-level methods were used for more effectively capturing the local structural changes in MRI scans. Zhu et al. (2021) have also proposed a method using image patch-level and multi-instance deep learning which achieves an accuracy of 90.2% for the AD classification task and 82.5% for the progression MCI task. Liu et al. (2019) extracted 27 overlapping 3D patches of size 50 × 41 × 40 voxels covering the whole volume of the MR image (100 × 81 × 80 voxels) then fit them into their model and achieve an accuracy of 93.7% in AD classification.

Although many studies have reported very high accuracy in AD classification task based on the deep learning model with neuroimaging data, there is currently a lack of studies regarding the prediction of MCI converting to AD. The progression of MCI classification has been challenging for not only computer-aid study but also clinical study. There is no obvious difference in brain anatomy between progression and stable MCI patients. Therefore, study about the progression of MCI classification using brain regions related to cognitive and sensory function is necessary.

Inspired by the success of transformers in Natural language processing (NLP), Dosovitskiy et al. (2021) developed the vision transformers (ViT) by formulating the image classification as a sequence prediction task for the patches. ViT and its variants have achieved SOTA performance on several datasets. Nowadays, transformers are becoming one of the most popular methodologies of computer vision tasks, including classification, detection, and segmentation. Coming up with many successes of vision transformers in the medical imaging field (Dai and Gao, 2021; Gao et al., 2021; Jun et al., 2021; Gheflati and Rivaz, 2022), we hypothesized that transformers also could advance the performance of MCI-to-AD progression.

The motivations of our proposed method are as follows:

1. Due to no obvious difference in brain atrophy among MCI patients, we hypothesize that the brain region responsible for cognitive and sensory function could be used as a good biomarker for computer-aid diagnosis in the progression of MCI. The mid-sagittal plane is used for MCI progression because it provides extremely good informative features of the mid-brain region, including the thalamus, medial frontal lobe, etc.
2. Vision transformers are proposed due to their successes in many medical classification studies before. We believe that ViT could replace other CNN-based methods in medical applications.

In this study, we proposed the first study to explore the potential of vision transformers in the medical image classification of MCI-to-AD progression by mid-sagittal planes MRI scan. We make the following contributions:

1. We apply vision transformers to sMRI classification of MCI progression for the first time and achieve state-of-the-art performance in respect of accuracy in comparison with recent studies.
2. We visualize the region that our model mostly focused on to ensure the interpretability and the reliability of our model. We found that the medial frontal and thalamus were strong predictors of MCI progression, in agreement with previous studies.

## 2. Materials and methods

### 2.1. Data sets and preprocessing

The sMRI scans used for this study are collected from the Alzheimer’s Disease Neuroimaging Initiative (ADNI^1^) database, including ADNI-1, ADNI-2, and ADNIGO. The ADNI was initiated in 2003 by Principal Investigator Michael W. Weiner, MD to test whether magnetic resonance imaging (MRI), positron emission tomography (PET), other biological markers, and clinical and neuropsychological tests can be incorporated to measure the development of MCI and early AD. We included all participants with a T1 weighted MRI scan at baseline from the ADNI1/GO/2: 258 MCI patients who progressed to AD within 36 months after the baseline time (MCIC) and 340 MCI patients who did not convert (MCINC). Table 1 shows the demographic details of subjects accessed from ADNI.

**Table 1:** Demographics information of the obtained subjects.

Diagnosis	Num. of Subjects	Gender (Female/Male)	Age (Mean ± Std)
MCIC	258	103/155	74.11 ± 6.99
MCINC	340	134/206	72.32 ± 7.48

**Figure 2:**
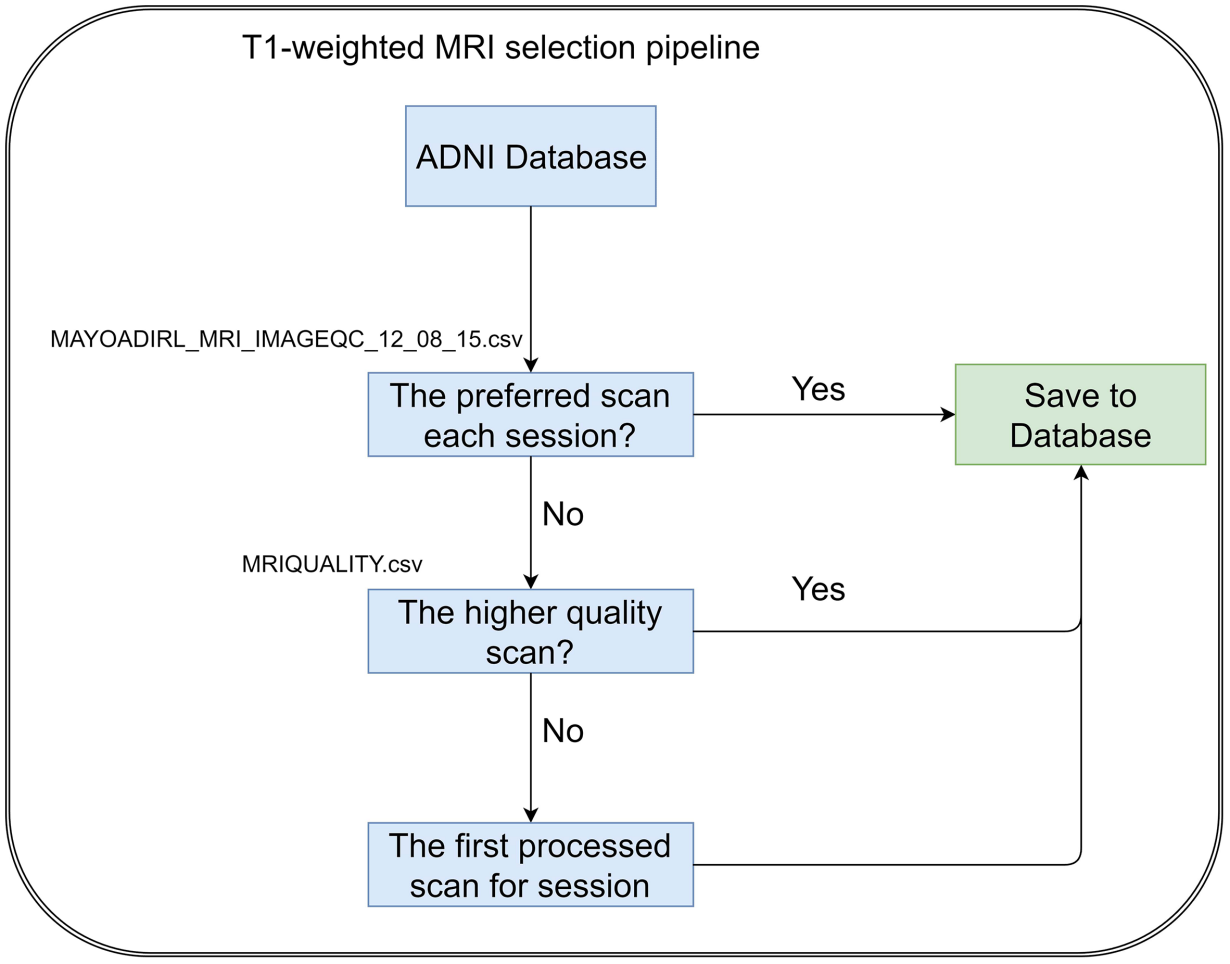
T1-weighted MRI scans selection.

The T1-weighted MRI scans were selected by following the steps, Figure 2. If there are multiple scans for a single session, we select the scan preferred by MAYOADIRL_MRI_IMAGEQC_12_08_15.csv, provided by ADNI. If no preferred scan is identified, we choose the higher-quality scan defined in MRIQUALITY.csv, also provided by ADNI. If there is no information regarding quality control, then we select the first scan for the visit.

The original sMRI data retrieved from the ADNI database are preprocessed to obtain improved image features for classification. In ADNI data, the scanners have different scanning parameters such as flip angle, slice thickness, etc. The ADNI scan intensity values were normalization by adjusting to have a zero mean and unit variance by subtracting the average intensity values and dividing the deviation. To reduce global linear differences or make all images fit into each other geometrically, we perform linear registration to the Colin27 templates (Holmes et al., 1998). Because the skull information is irrelevant for MCI progression prediction, skull-removing is applied for all images. Registration and skull-stripping steps were performed by ‘FLIRT’ function with default parameter and ‘BET’ function with fractional intensity threshold (0.5) of the FSL toolbox (Jenkinson et al., 2012) respectively. After preprocessing, the MR images have a size of 181 × 217 × 181. Then we extract three middle slices of sagittal planes (middle ±3 voxels) to form mid-sagittal planes with the size of 3 × 217 × 181. The mid-sagittal images then are resized to 3 × 224 × 224 in order to fit the pre-trained model. The zero-padding was applied to resize for keeping the same resolution. Figure 2 shows the pipeline of our preprocessing process.

### 2.2. Vision transformers architecture

This work is oriented towards the exploitation of Vision Transformers (ViT) approaches. Proposed by Dosovitskiy et al. (2021) the ViT is an architecture for image classification that employs a Transformer-like architecture over patches of the image and can outperform common CNN architectures when trained on large amounts of image data. The concept of vision transformers is described as follows:

A standard transformer receives an input as a 1D sequence of token embeddings; therefore in order to handle a 2D image, ViT reshapes the image $I\epsilon \;R^{H\times W\times C}$ into a sequence of flattened 2D patches $I^{P}\epsilon \;R^{n\times \left(P^{2}\times C\right)}$ , where (H,W) is the resolution of the original image, C is the number of channels, P is the resolution of image patch and $n=HW/P^{2}$ is the number of patches. Vision transformers flatten the patches and transform image patches to a D dimension vector with a trainable linear projection because vision transformers use the same width across all layers. The output of this projection is used as the patch embeddings.

The essential components of the standard transformer layers include Multi-Head Self Attention (MSA) and Multi-Layer Perceptron (MLP). The multi-head self-attention mechanism splits the input into many small parts, then measure the scaled dot-product of each input in parallel, and splices all the attention outputs to have the final outputs of multi-head self-attention:


(1) $$Attention\left(Q,K,V\right)=Softmax\left(\frac{QK^{-T}}{\sqrt{d_{x}}}\right).V$$

(2) $$head_{i}=Attention\left(QW_{i}^{-Q},KW_{i}^{-K},VW_{i}^{-V}\right)$$

(3) $$MSA\left(Q,K,V\right)=Concat\left(head_{i},\ldots ,head_{i}\right)W^{O}$$

The multi-layer perceptron is added on top of the MSA layer. The MLP module contains linear layers separated by a Gaussian Error Linear Unit (GeLU) activation. Both MSA and MLP have skip-connection-like residual networks and layer normalization.


(4) $$x_{t}^{\prime }=MSA\left(LN\left(x_{t-1}\right)\right)+x_{t-1}$$

(5) $$x_{t}=MLP\left(LN\left(x_{t}^{\prime }\right)\right)+x_{t}^{\prime }$$

Where $x_{t-1}$ represent for the t−1 layer, LN represents the linear normalization, $x_{t}$ is the output of the t-th layer.

### 2.3. Our methodology

**Figure 3:**
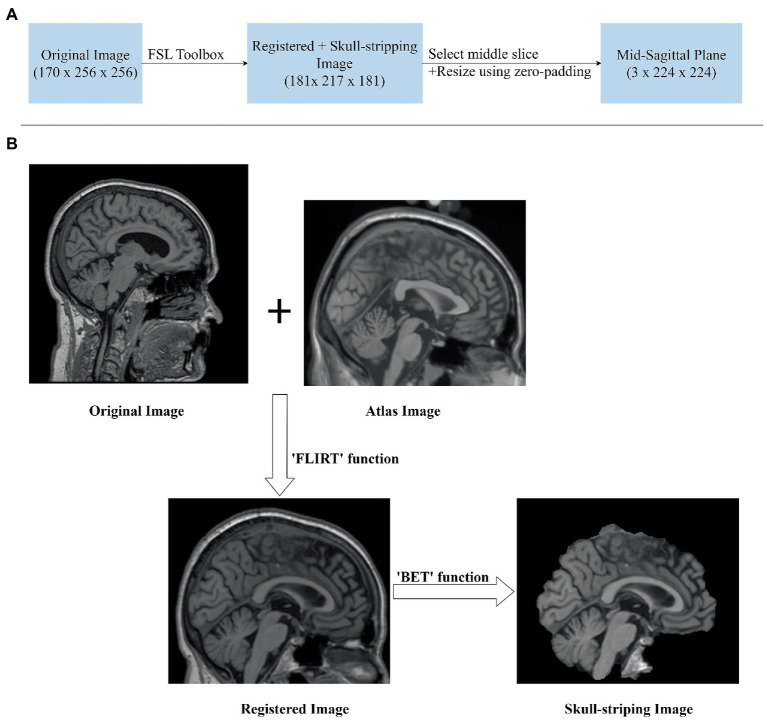
sMRI Image preprocessing. **(A)** Image preprocessing pipeline for extracting mid-sagittal plane and fit to the model. **(B)** Sagittal plane view of the image in original, registration, and skull-stripping stages.

In this study, we followed the original ViT implementation as much as possible to intuitively compare the benefits of transformers and access the extensible ViT model and its pre-trained almost immediately. The structure of our model is shown in Figure 3.

Given original MR images $I\epsilon \;R^{B\times Ch\times S_{original}\times C_{original}\times A_{original}}$ , where $S_{original}\times C_{original}\times A_{original}$ (170 × 256 × 256 voxel) is sagittal, coronal and axial spatial resolution, the number of channels is Ch , the batch size is *B*. After registration and skull-stripping, the image will become $I_{preprocessed}\epsilon \;R^{B\times Ch\times S_{new}\times C_{new}\times A_{new}}$ , where $S_{new}\times C_{new}\times A_{new}$ (181 × 217 × 181 voxel) is the new sagittal, coronal, axial spatial resolution after preprocessing. Before sending it to transformers, it is necessary to extract mid-sagittal slices from the preprocessed images to 2D mid-sagittal slices images $I_{mid-sagittal}\epsilon \;R^{B\times 3\times C_{final}\times A_{final}}$ , where $C_{final}\times A_{final}$ is the final spatial resolution. Here, we choose three mid-sagittal slices (mid slices ±3 voxels) of the sagittal plane to form three channels of a 2D image. Then, the image sequences are constructed and fit to the model.

### 2.4. Experimental setting and evaluation criteria

Our total dataset has 2,681 images from 258 MCIC and 340 MCINC patients. We shuffled the images randomly and all experiments were performed by splitting data into 10% as test and 90% as train data; 20% data from the train set is used as a validation set. We set SGD as the optimizer with a learning rate equal to $1e^{-7}$ . The model is trained for 200 epochs and an L2-Regularization value of $1e^{-5}$ . Our experiments are done on a machine with an Intel(R) Xeon(R) Gold 6258R CPU @ 2.70GHz with 256GB RAM. The GPU used is 4x NVIDIA GeForce RTX 3090. The code is implemented using Pytorch (Paszke et al., 2019) and PyCharm (PyCharm, n.d.).

The evaluation criteria for the model are accuracy rate (ACC), sensitive rate (SEN), and specificity (SPEC), defined as follows:


(6) $$ACC=\frac{TP+TN}{TP+TN+FP+FN}\;$$

(7) $$SEN=\frac{TP}{TP+FN}$$

(8) $$SPEC=\frac{TN}{TN+FP}$$

where TP, TN, FP, and FN denoted as true positive, true negative, false positive and false negative value, respectively.

## 3. Results and analysis

### 3.1. Classification performance on ADNI

**Figure 4:**
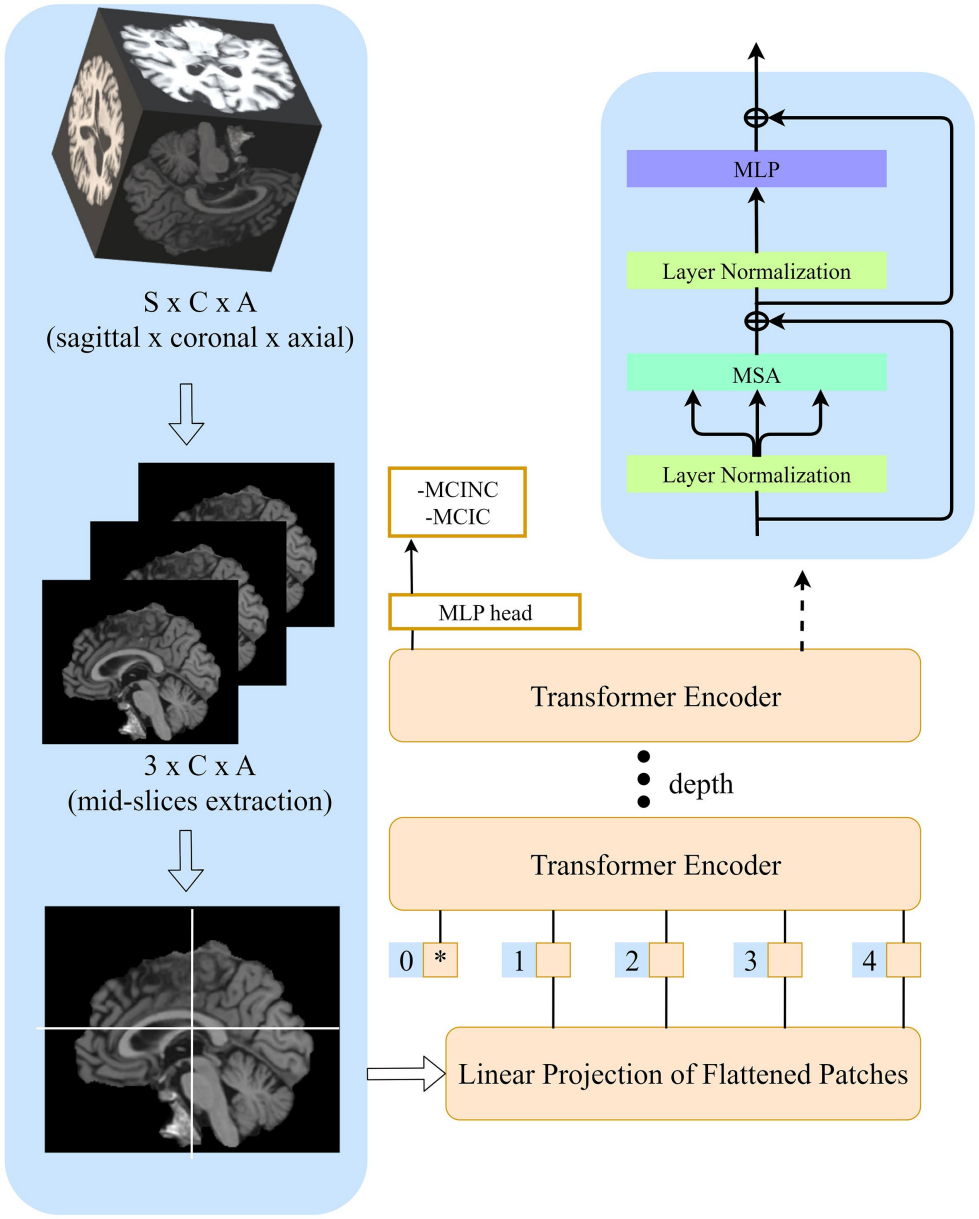
Overview of sMRI-based vision transformers model in prediction of MCI-to-AD progression.

The performance on MCI-to-AD progression prediction achieved by mid-sagittal MRI-based vision transformers is shown in Table 2 and Figure 4. The tables include our results and those of the literature. For each study, we indicate the methodology, and the values of the performance measures. We provided three variants of our method: the small version (VIT-S), the base version (VIT-B), and the large version (VIT-L) of vision transformers. Our method consistently outperforms previous studies in three indicators of classification performance including sensitivity (Sen), specificity (Spec), and accuracy (Acc). The VIT-S archives 83.27% in accuracy and 85.07% which is about a 1 and 4% improvement, respectively in comparison with the other studies (Eskildsen et al., 2013; Basaia et al., 2019; Bae et al., 2021; Zhang et al., 2021; Zhu et al., 2021; Ashtari-Majlan et al., 2022). VIT-B also shows a significant performance enhancement in specificity with 82.22%. Although Ashtari-Majlan et al. (2022) have shown higher specificity (99.70%), their results are lower in both accuracy and sensitive indicators. Figure 4 shows the confusion matrix of the VIT-S model which has the best result among our methods with an AUC of 0.87. Finally, our proposed method is highly efficient compared with state-of-the-art MRI-based research.

**Table 2:** Referential comparison between the proposed model with MRI-based studies for MCI progression prediction (in %).

Study	Method	Acc	Sen	Spec
Basaia et al. (2019)	3D CNN architecture	75.1	74.8	75.3
Bae et al. (2021)	CNN with ResNet backbone	82.4	81.08	–
Zhang et al. (2021)	Connection-wise-attention-model-based densely connected convolution neural network (CAM-CNN)	78.79	75.16	82.42
Eskildsen et al. (2013)	mRMR, linear discriminant analysis (LDA)	77.30	69.00	79.10
Ashtari-Majlan et al. (2022)	Multi-stream convolutional neural network	79.90	75.55	99.70
Zhu et al. (2021)	Dual attention multi-instance deep learning network	80.20	77.10	82.60
VIT-S	Vision transformers	83.27	85.07	81.48
VIT-B	Vision transformers	80.67	79.10	82.22
VIT-L	Vision transformers	72.86	74.63	71.11

### 3.2. Pathological locations attentions by transformers

**Figure 5:**
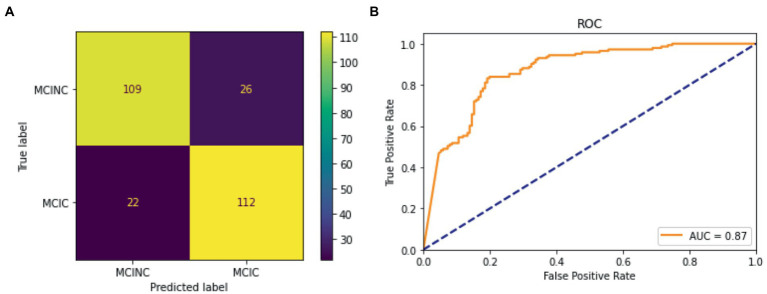
Classification result for the proposed model for MCINC and MCIC class. **(A)** Confusion matrix of the test dataset. **(B)** ROC curve (receiver operating characteristic curve).

Defining the brain region most related to the deep learning model prediction is of great importance to computer-aided diagnosis. One of the keys to the clinical diagnosis of AD and the progress of MCI to AD is to observe the structural change in the brain. As a prediction approach, we investigate the possible pathological region in the brain which is related to the prediction of our method. We use GRAD-CAM (Selvaraju et al., 2020) to investigate which brain region attention layers observe and focus on in order to classify MCIC and MCINC classes (Figure 5). depicts several locations in mid-sagittal slices identified by our proposed method. The marked locations are, respectively, suggested by attention score using GRAD-CAM. In the left panel, we compare the MCIC and MCINC classes in each marked location. The right panel shows the related brain regions of marked locations. We find that three major regions that are most informative for our model prediction are the thalamus, medial frontal, and occipital. We observed brain atrophy in the medial frontal and occipital of MCIC class compared with these of MCINC. The right panel shows the related brain regions of marked locations. These marked regions are consistent with many previous studies about AD diagnosis (Lian et al., 2020; Shao et al., 2020; Bron et al., 2021) - attesting the reliability of the proposed model.

### 3.3. Ablation experiment

To investigate the effect of design choices for transformers in MCI-to-AD classification, we conducted an ablation experiment. In the experiment, we explore the impact of different models, the size of ViT, patch size, and pre-trained weight. We experimented in 3 versions of our method: small version (VIT-S), base version (VIT-B), and large version (VIT-L). All models were trained using pre-trained weights available in Pytorch-Image-Model implementation (Wightman et al., 2022), including ImageNet-1K, ImageNet-21K, and Facebook DINO pre-trained weight. Patch size was selected between 8, 16, and 32. The results are shown in Table 3. We observe that the ViT small version with a patch size of 16 and pre-trained weight of ImageNet-1K gives the best classification performance. From our point of view, the patch size of 16 could catch the most effective and informative features of MR images. The proposed model makes predictions by extracting the brain regions with related patch sizes. With the larger patch size, the information collected by the model becomes too generalized and loses a lot of details leading to underfitting. In contrast, too small an image patch size can destroy the semantic information of the MRI scan.

**Table 3:** Ablation study on the effectiveness of transformers.

Model	Patch size	Pretrained data	Accuracy	Sensitivity	Specificity
VIT-B	32	ImageNet-1k	76.21	77.04	75.37
		ImageNet-21k	75.84	75.56	76.12
	16	ImageNet-1k	80.67	82.22	79.10
		ImageNet-21k	76.95	75.56	78.36
		Facebook DINO	80.67	82.22	79.10
	8	ImageNet-1k	73.98	72.59	75.37
		ImageNet-21k	72.86	71.11	74.63
		Facebook DINO	81.41	82.96	79.85
VIT-S	32	ImageNet-1k	73.61	71.85	75.37
		ImageNet-21k	75.84	72.59	79.10
	16	ImageNet-1k	83.27	81.48	85.07
		ImageNet-21k	76.58	76.30	76.87
		Facebook DINO	73.98	69.63	78.36
	8	Facebook DINO	77.32	71.85	82.84
VIT-L	32	ImageNet-21k	72.49	69.63	75.37
	16	ImageNet-1k	72.86	71.11	74.63
		ImageNet-21k	71.38	69.63	73.13
	8	ImageNet-1k	74.35	71.11	77.61
		ImageNet-21k	73.61	71.85	75.37

## 4. Discussion

Effective and precise MCI-to-AD prediction is critical for early intervention and management of the disease. Therefore, many studies make an effort to research and improve the performance of MCI progression prediction. In this study, we investigated a comparative study focusing on the predictive performance of vision transformers based on mid-sagittal slices sMRI data of the ADNI. Our proposed method outperformed the current state-of-the-art MRI-based studies on MCI progression diagnosis (Basaia et al., 2019; Bae et al., 2021; Zhu et al., 2021; Ashtari-Majlan et al., 2022) with an accuracy of 83.27%, specificity of 85.07%, and sensitivity of 81.48%. These results imply that using vision transformers equipped with attention power could achieve better classification performance compared with the current CNN architecture. The possible reason is that the attention mechanism in vision transformers could effectively enhance the difference in the brain region between MCI convert and no convert classes.

In the ablation contribution, we conducted the variant of the model, patch size, and pre-trained weights to better understand the efficacy of the proposed method. For patch size, we observe that the proposed method could gather better informative features with a patch size of 16. The results also have shown that reducing the complexity of the model leads to better accuracy, where ViT-S gives us the best accuracy. We assume that the reason is the poor performance of the pure transformer with larger complexity due to the small dataset. These also are proven by the better results of Facebook DINO pre-trained weight in the base version (VIT-B). DINO Facebook pre-trained is the weight trained by self-distillation with no label’s methods (Caron et al., 2021). Through the distillation train approach, their model has worked efficiently even with a small dataset. In future work, we will explore the performance of the self-distillation train approach in our proposed method.

**Figure 6:**
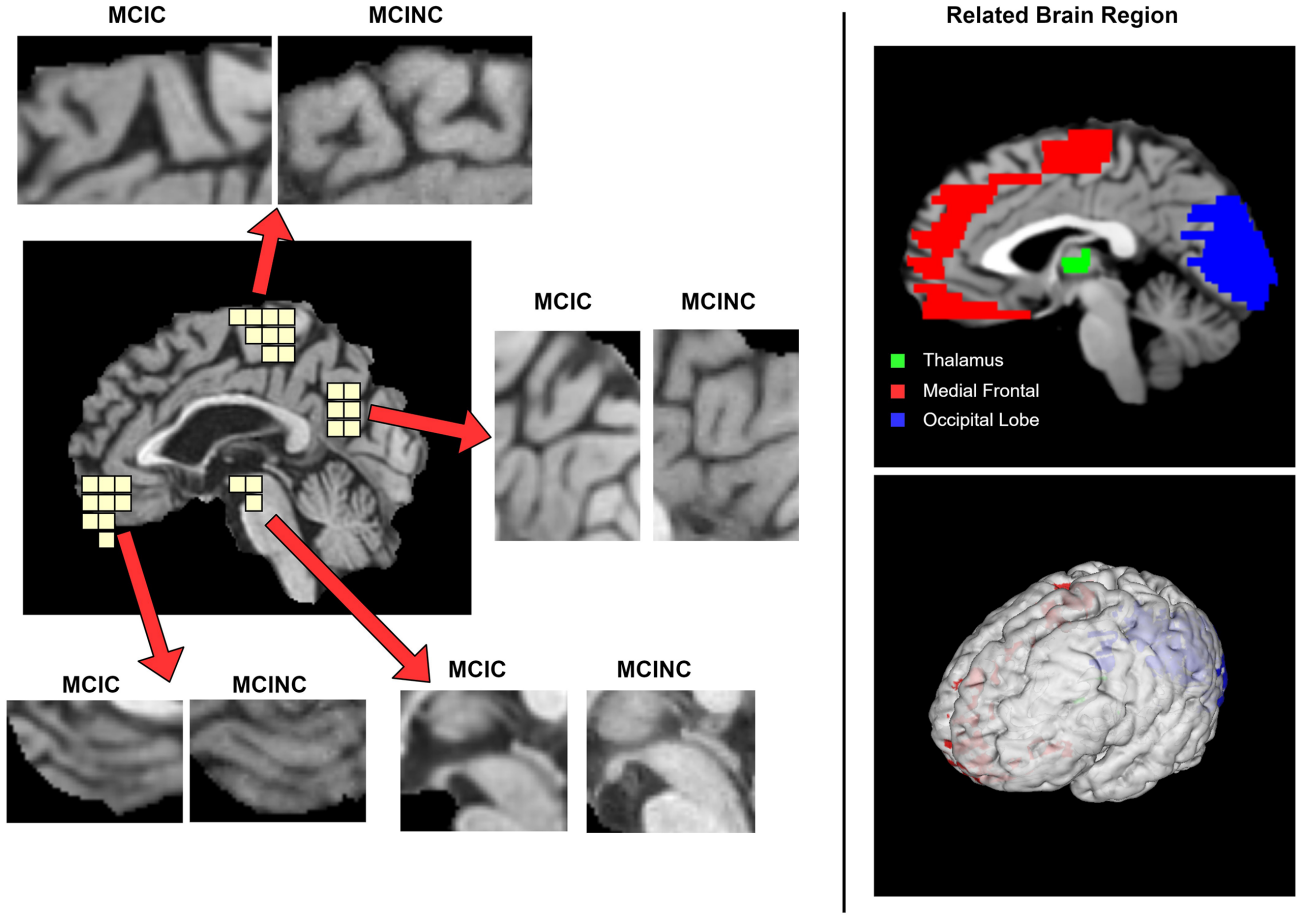
Visualization of the pathological sagittal brain region identified by proposed method on MCI-to-AD Progression Classification. The **left** panel shows the informative locations suggested by attention scores and the comparison between MCIC and MCINC classes in each region. The **right** panel shows the related brain region, respectively, with marked locations.

**Figure 7:**
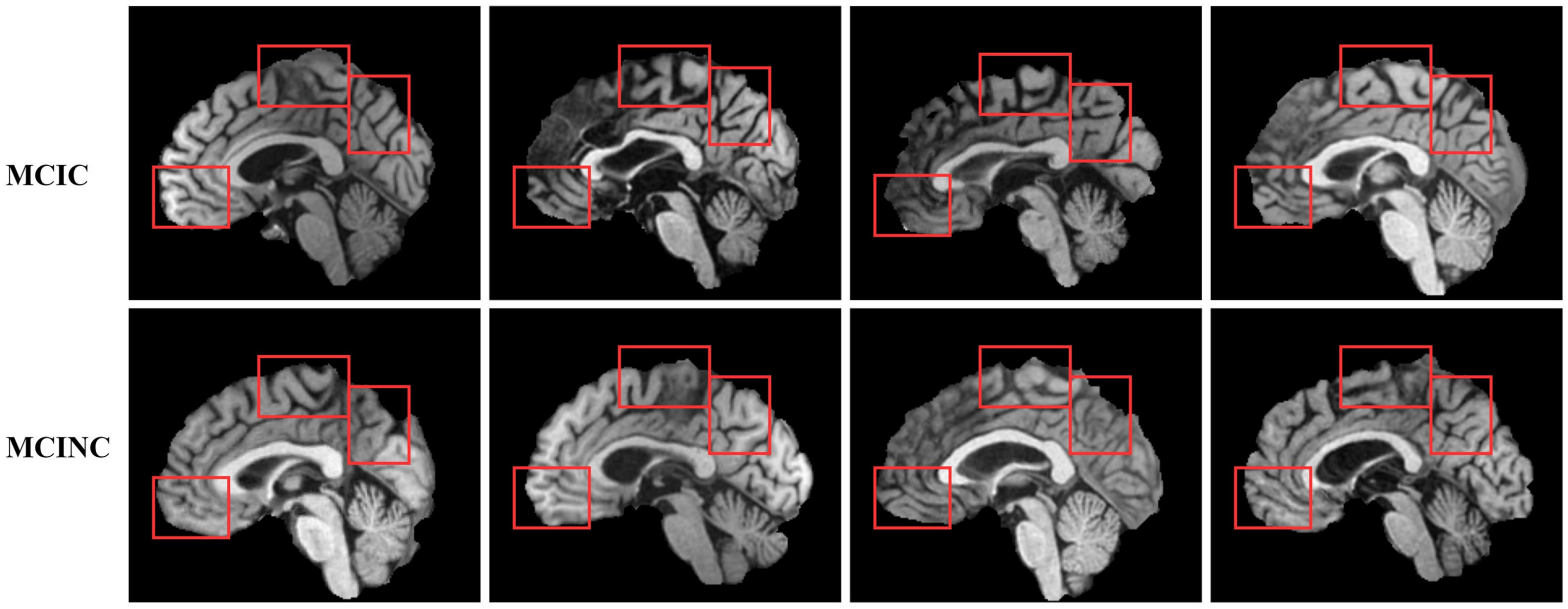
Example of MCIC and MCINC MRI scans with region of interest (ROI).

Besides, we also observed the brain regions that affect our proposed method’s prediction. Identifying these regions will help for future development of deep learning models to improve classification performance as well as for doctors to find the regions of interest for diagnosis easily. We mark out three main regions that have the highest attention score: the thalamus, medial frontal, and occipital. We also observed brain atrophy in these regions in MRI scans. Figure 6 have shown examples of MRI scans in both convert (MCIC) and non-convert (MCINC). Specifically, the thalamus is the main relay of sensorimotor information in the brain and is thought to be crucial for memory processing which is impacted early in Alzheimer’s disease (Aggleton and Brown, 1999; Aggleton and Nelson, 2015). The medial frontal also plays an important role in numerous cognitive functions, including attention, and spatial or long-term memory (Jobson et al., 2021; Park et al., 2021). The occipital is responsible for visual perception, including color, form, and motion where the volume is reduced due to Alzheimer’s disease (Holroyd et al., 2000; Brewer and Barton, 2014). These results suggest informative regions for future feature extraction to improve our proposed method by focusing more attention on these locations. In addition, the three marked brain regions which contribute critical information for the prediction of the method also give more useful clues for doctors in clinical diagnosis (Figure 7).

## 5. Limitations and future work

Although our proposed method achieves good performance in AD-related diagnosis, several limitations still need to improve in the future. We summarize the limitations and potential solutions as follows.

Our method is not actually a 3D scan model, only 3 slices of the mid-sagittal brain are extracted. Therefore, the global anatomical information in another brain region could be missed during the prediction. In Alzheimer’s disease diagnosis, the hippocampus and media temporal lobe is critical regions. Missing the image slices covering these regions could lead to misdiagnosing the disease. The segmentation of these regions is needed in future work.

Moreover, the 3DViT consumed a lot of computational time when trained with 224 × 224 × 224 images. In future work, embedding only the attention mechanism of vision transformer into convolution neutral network model could be one of the solutions to reduce the computational time when performing the model in the 3D scan. The automatic segmentation of the hippocampus and media temporal lobe from a 3D scan could reduce the image size and keep the necessary information for disease diagnosis.

## 6. Conclusion

In this work, we proposed a vision transformers model to advance the classification accuracy in MCI-to-AD conversion prediction, which includes two major contributions:

1. Our proposed method is evaluated on 598 subjects from ADNI datasets. As far as we know, we are the first study to develop a ViT model for midsagittal sMRI for MCI to AD progression classification. We achieved a classification accuracy of 83.27%, specificity of 85.07%, and sensitivity of 81.48%. Compared with other MRI-based studies on the same datasets, the proposed method has demonstrated top-ranked classification accuracy.
2. We visualized the brain regions affected mostly to the performance of our method. We found that the thalamus, medial frontal, and occipital regions of sMRI were the strongest features in our proposed model. These results highlight the potential for early diagnosis and stratification of individuals with MCI based on patterns of cortical atrophy, prior to interventional clinical trials.

## Data availability statement

The raw data supporting the conclusions of this article will be made available by the authors, without undue reservation.

## Ethics statement

Written informed consent was obtained from the individual(s) for the publication of any potentially identifiable images or data included in this article.

## Author contributions

GH: experiments, data analysis, and manuscript writing. JK: conceptualization, designing experiments, supervision, review and revision of the manuscript. U-HK: conceptualization, designing experiments, supervision, fund acquisition, review and revision of the manuscript. All authors contributed to the article and approved the submitted version.

## Funding

This work was supported by Healthcare AI Convergence Research & Development Program through the National IT Industry Promotion Agency of Korea (NIPA) funded by the Ministry of Science and ICT (No. S1601-20-1016); National Research Foundation of Korea (NRF-2022R1A2C3009749) and “GIST Research Institute (GRI) IIBR” grant funded by the GIST in 2023.

## Conflict of interest

The authors declare that the research was conducted in the absence of any commercial or financial relationships that could be construed as a potential conflict of interest.

## Publisher’s note

All claims expressed in this article are solely those of the authors and do not necessarily represent those of their affiliated organizations, or those of the publisher, the editors and the reviewers. Any product that may be evaluated in this article, or claim that may be made by its manufacturer, is not guaranteed or endorsed by the publisher.

## Footnotes

^1^ adni.loni.usc.edu/

## Abbreviations

AD: Alzheimer’s disease
MCI: mild cognitive impairment
CNN: convolutional neural network
ViT: vision transformers
sMRI: structural magnetic resonance image
MCINC: non-convert MCI
MCIC: convert MCI
NLP: natural language processing
PET: positron emission tomography
MLP: multi-layer perceptron
